# B-Flow Twinkling Sign in Preoperative Evaluation of Cervical Lymph Nodes in Patients with Papillary Thyroid Carcinoma

**DOI:** 10.1155/2013/203610

**Published:** 2013-06-26

**Authors:** Giuseppina Napolitano, Antonio Romeo, Andrea Bianco, Maurizio Gasperi, Pio Zeppa, Luca Brunese

**Affiliations:** ^1^Department of Health Science, Chair of Radiology, University of Molise, Contrada Tappino, 86100 Campobasso, Italy; ^2^Department of Medicine and Surgery, the University of Salerno (IT), Italy

## Abstract

Papillary thyroid cancer (PTC) is the most common histologic type of differentiated thyroid cancer. The first site of metastasis is the cervical lymph nodes (LNs). The ultrasonography (US) is the best diagnostic method for the detection of cervical metastatic LNs. We use a new technique, B-flow imaging (BFI), recently used for evaluation of thyroid nodules, to estimate the presence of BFI twinkling signs (BFI-TS), within metastatic LNs in patients with PTC. Two hundred and fifty-two patients with known PTC were examined for preoperative evaluation with conventional US and BFI. Only 83 with at least one metastatic LN were included. All patients included underwent surgery; the final diagnosis was based on the results of histology. The following LN characteristics were evaluated: shape, abnormal echogenicity, absent hilum, calcifications, cystic appearance, peripheral vascularization, and BFI-TS. A total of 604 LNs were analyzed. Of these, 298 were metastatic, according to histopathology. The BFI-TS showed high values **of specificity (99.7%) and sensitivity (80.9%). The combination of each conventional US sign with the BF-TS increases the specificity. Our findings suggest that BFI can be helpful in the selection of suspicious neck LNs that should be examined at cytologic examination for accurate preoperative staging and individual therapy selection.

## 1. Introduction

Papillary thyroid carcinoma (PTC) is the most common histologic type of differentiated thyroid cancer and accounts for 80% of all thyroid cancers [1, 2]. The disease-specific survival rate of PTC is excellent but its recurrence rate is high [2–4]. PTC and the follicular variant of PTC have a propensity for cervical lymphatic spread that occurs in 20% to 50% of patients on standard review of surgical pathologic specimens and in 90% of those examined for micrometastases [3–7]. The spread of tumor cells occurs in a predictable pattern that initiates in the perithyroidal lymph nodes (LNs) of the central neck and progresses to the LNs of the lateral cervical compartments and the superior mediastinum [8, 9]. Skip metastases to the lateral compartment without central neck nodal involvement are rare but do occur [8, 9]. Patients with nodal metastasis have higher rates of persistent and recurrent disease during postoperative surveillance [10]. Furthermore, lymph node metastasis has also been identified as a risk factor for distant metastasis.

Several studies have shown that ultrasonography has higher sensitivity than palpation and the other diagnostic methods for the detection of cervical metastatic LNs in patients with PTC [11]. Ultrasound is easily repeatable and has been shown to change the surgical procedure performed in 39% of thyroid cancer patients. [12, 13]. Metastatic LNs tend to be large, round, hypoechoic, and hypervascularized with a loss of hilar architecture [14, 15]. In differentiated thyroid cancer, metastatic LNs may also have specific features such as hyperechoic punctuations or microcalcifications and cystic appearance [16–18].

B-flow imaging (BFI) is a non-Doppler technique widely used to evaluate carotid artery stenosis and other vascular diseases [19]. The BFI technique has recently been used to evaluate thyroid nodules [20, 21]. BFI can identify a new sign (the twinkling sign; BFI-TS) in “suspect” PTC nodules, which appeared to be generated by microcalcifications and increase the US accuracy in identification of malignant nodules [20, 21]. The BFI-TS is a rapidly flashing white light behind such stationary objects as microcalcifications, which gives the appearance of movement.

When an incidental sonographic beam impinges a rough interface composed of sparse reflectors, the sign is generated by the phase shift, thereby causing a faint variation of the sonographic beam at the interface. The sign is also caused by the increase of pulse duration, which results in multiple reflections in the medium. In thyroid nodules, these rough interfaces were the microcalcifications formed from aggregates of primary psammoma bodies (PBs); they consist mainly of highly reflecting crystalline aggregates of calcium [22]. The same features described in thyroid nodules, represented by microcalcifications and colloid crystals, are also present in lymph node metastases. The aim of this study was to determine the presence of BFI-TS in metastatic LNs and to compare it with the other ultrasound features in relation to the results obtained from the surgical specimen. 

## 2. Materials and Methods

### 2.1. Patients

Between September 2006 and December 2011, 252 patients with known PTC were examined at our institution for preoperative sonographic evaluation with grayscale ultrasonography (US), color Doppler US, and BFI-TS. Moreover, 121 patients with suspicious metastatic cervical LN at US examination underwent FNAB for cytology and thyroglobulin determination in the aspirate fluid. Only 83 patients (19 men, 64 women; mean age 52 years range, 26–79 years) with at least one metastatic LN were included in our study. All these patients underwent surgery, and the final diagnosis was based on the results of histologic examination of the resected specimens. The mean interval between sonographic examination and surgery was 5.3 days (range 1–17 days). The study was conducted at the Department of Radiology and Endocrinology of the University of Naples Federico II and at the Department of Endocrinology of the Second University of Naples, according to the principles of the Declaration of Helsinki and approved by the Ethics Committee of the University of Molise. Written informed consent was obtained from all subjects.

### 2.2. US and Cytological Examinations

US, color Doppler, and BFI examinations were performed with LOGIQ 9 GE Healthcare (Chalfont St Giles, UK), a commercially available real-time US system, equipped with a 5 to 14 MHz (M12L) and 2.5 to 7 MHz (7L) linear array transducer. All examinations were performed by two blinded radiologists with 8 and 10 years of neck sonography experience separately, and all data analysis was performed by another investigator. When results of the examiners were discordant, agreement was found by conjoint review of clips of the US examinations. At grayscale US, the following six sonographic characteristics were evaluated for all LNs examined: a round shape (ratio of short axis to long axis >0.5), absence of echogenic hilum, abnormal echogenicity of LN, calcification, cystic change, and a peripheral color Doppler pattern. The shape, size, and location (levels I–VI) of all cervical LNs were recorded, based on the American Joint Committee on Cancer and the American Academy of Otolaryngology-Head and Neck Surgery nodal classification [23–25].

BFI was performed at 10 MHz (M12L) and 7 MHz (7L) with the BFI capability at the level of the LNs. PRI was set at 3. BFI gain was not fixed and was adjusted to allow a better visualization of the signs. This technique focuses on high flow, with suppression of the tissue signal. BFI images were used to evaluate the presence or the absence of the signs. The BFI-TS is a rapidly flashing white light behind such stationary objects as microcalcifications and colloidal crystals. The sign was considered positive when at least a twinkling was present in the LNs examined and repeatable over time.

After the US features were assessed, patients underwent a cytological evaluation. US-guided FNA was simultaneously performed by an endocrinologist, a radiologist, and pathologist. Physicians were highly experienced in carrying out US-guided FNA using 27- and 22-gauge needles; the technique used is described elsewhere [26, 27]. Three or four smears were prepared; the first was air dried and immediately stained with Diff Quick stain. Inadequate smears were immediately repeated. After collection of the cytology samples, each FNAB needle was washed with 0.1–0.5 mL of normal saline; the washes from all needles were pooled (final volume 0.5–1 mL) and sent to the laboratory. Thyroglobulin was measured in fine needle washouts using an immunoradiometric assay (IRMA—DYNOtest Tg-plus, BRAHMS Diagnostica GmbH, Berlin, Germany).

When the measured FNAB-Tg level was greater than the serum Tg level, we deemed the LN positive for metastasis from PTC.

### 2.3. Surgery and Histologic Examination

All patients underwent thyroidectomy and ipsi- or bilateral modified radical neck dissection to include levels II–V. All possible measurements were taken to ensure an accurate one-to-one comparison between the LNs that were imaged and those that were removed during surgery. After US examination, the location of each lymph node was mapped with respect to the surrounding anatomic structures (i.e., trachea, main vessels, and sternocleidomastoid muscle) and plotted on the sketched diagram of the neck. Surgeons were assisted by a radiologist for correlation of the LN location seen on the US images with the LNs seen in the lymphadenectomy specimens. After being resected, each LN specimen was fixed in 10% formalin, embedded in paraffin, cut into thin slices, and stained with standard hematoxylin-eosin. During histologic examination, two or three histologic slices per LN were examined. The final diagnosis of metastatic lymph node involvement was made by a pathologist who had 15-year experience in diagnosing histologic cervical LN. Complete versus incomplete metastatic involvement and the presence of necrosis and/or calcifications were also investigated.

### 2.4. US and Pathology Correlation

To match each LN found at pathological examination to the corresponding node on US, we took into account its location, shape, and size. Only LNs that were unequivocally matched between US and pathology were taken into account. Multiple LNs at a given neck level on US were taken into account only if all LNs of the compartment were either benign or malignant.

### 2.5. Statistical Analyses

Qualitative variables were compared by using the *χ* ^2^ test. The BFI characteristics of each LN were recorded separately and processed blindly for statistical evaluation. The unit of analysis was each LN rather than each patient. The value of each visual and qualitative criterion that showed the highest diagnostic accuracy in the distinction between benign and metastatic lymph nodes was selected as the cutoff value. For each criterion examined, the sensitivity, specificity, positive and negative predictive values, and overall accuracy in the differentiation between benign and metastatic LNs were calculated. Quantitative data are reported as means ±1 standard deviation. Statistical significance was assumed when the *P* value was less than 0.05. The same analysis has been performed on the association between the BFI and each ultrasound parameter. 

## 3. Results

A total of 604 LNs were analyzed. Of these, 298 were metastatic while the remaining 306 were benign, as evaluated by histopathology. The minimum diameters of LNs on sonography ranged from 2.3 to 13 mm; the mean diameter of metastatic LNs was 5.8 mm, and the mean diameter of nonmetastatic LNs was 4.6 mm; the difference was not significant (*P* > 0.05). The diagnostic performance of each ultrasound finding evaluated in this study is shown in Table 1. Most ultrasound features had high specificity and positive predictive value (PPV) but low sensitivity and negative predictive value (NPV). The only sonographic characteristic with high specificity and sensitivity was the BFI-TS. The BFI-TS was positive in all LNs with microcalcifications at US examination (93 LNs) and in 148 LNs (all metastatic) in which microcalcifications were not evident at US. One LN positive at the BFI-TS and with calcifications at US was found to be a tuberculous node after treatment with intranodal macrocalcifications at histological examination.

The diagnostic performance of the combination of each conventional ultrasound sign with the BFI-TS is shown in Table 2. This combination allowed to increase the specificity and the PPV related to different ultrasound signs. The association of the absence hilum with BFI-TS presented the highest values of sensitivity, specificity, and PPV. 

## 4. Discussion

**Figure 1:**
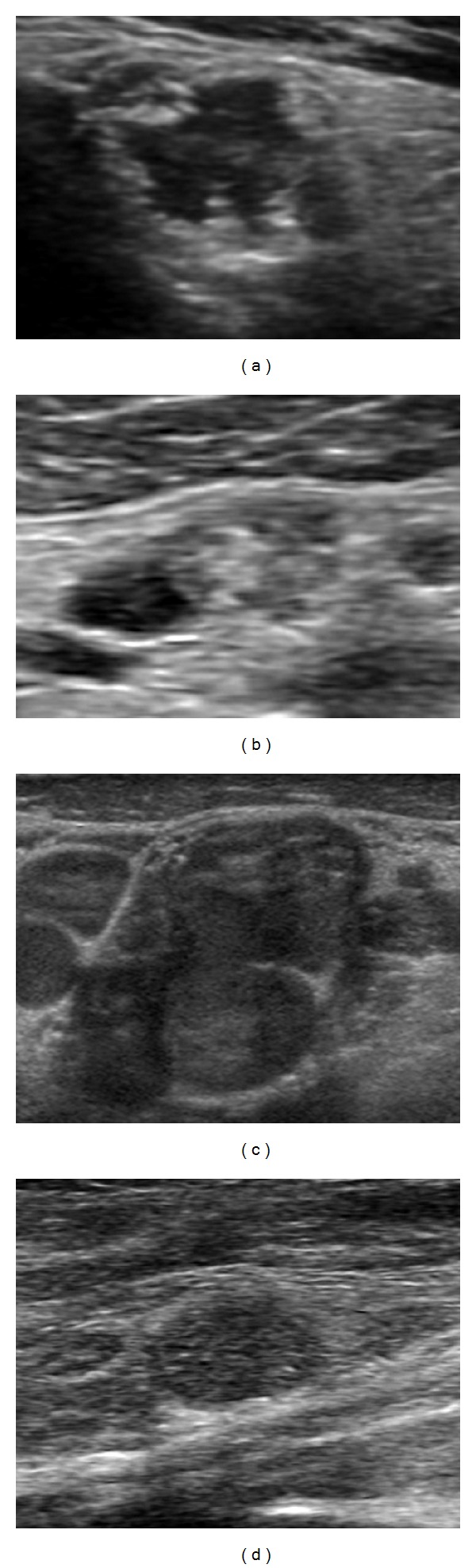
Metastatic lymph nodes at grayscale examination in patients with papillary thyroid cancer. Absence of echogenic hilum ((a), (b), (c), (d)), abnormal echogenicity ((a), (b), (c)), calcifications (b), cystic change ((a), (b)), and round shape ((c), (d)).

Neck US is highly sensitive for the diagnosis of metastatic LNs in patients with PTC. The specificity reported varies from 85% to 90% [28]. A variety of diagnostic criteria have been reported to be useful for the distinction between benign and metastatic LNs (Figure 1).

*Lymph node shape* has been used as a diagnostic criterion of metastatic LNs. Metastatic lymph nodes often appeared as round lesions, whereas benign nodes are usually flat or oval [29]. In the present study, LN shape had an excellent specificity (90.8%) but low sensitivity (52%). Initial or partial metastatic LN involvement does not result in an alteration of the shape. Of note, LNs of the parotid and submandibular regions are often round in normal individuals [30].

*The presence of a hyperechoic hilum* of the nodes is usually considered a strong diagnostic criterion for benign LNs [31]. It has been reported that 84%–92% of benign nodes but less than 5% of metastatic nodes have a hyperechoic hilum [32]. The absence of a fatty hilum is often seen in normal individuals, especially in young subjects and in LNs located in level V [33]. In our study, metastatic LNs with visible hilum and partial involvement were at I-II level, whereas the LN metastases at low level showed in 99.5% the absence of hyperechoic hilum. The absence of the hilum had high sensitivity (91.9%) but low specificity (58.8%). Differently, *abnormal LN echogenicity* had both high sensitivity and specificity (resp., 81.9% and 85%). In our experience, echogenicity was normal in 54 metastatic LNs (18%). *Calcification* was a specific sign but not sensitive criterion. Calcification in metastatic LNs is characteristic of PTC but generally rare. In our study, nodal calcifications were detected in only 93 of the 298 metastatic LNs. Similarly, *cystic appearance* had a very high specificity (100%) and a low sensitivity (21.1%). All LNs with hyperechoic punctuations or a cystic appearance in a patient w *i*th PTC should be considered as malignant. *Assessment of nodal vascularity* at color Doppler US is another diagnostic criterion for metastatic LNs. It has been noted that benign LNs tend to show hilar vascularity or to appear avascular [34]. In contrast, metastatic nodes tend to have peripheral or mixed (both peripheral and hilar) vascularity [35]. In our study, color Doppler US vascularity had intermediate specificity (68.6%) but low sensitivity (47.6%). These findings could reflect the high differentiation of PTC and the reduced tendency to neoangiogenesis.

**Figure 2:**
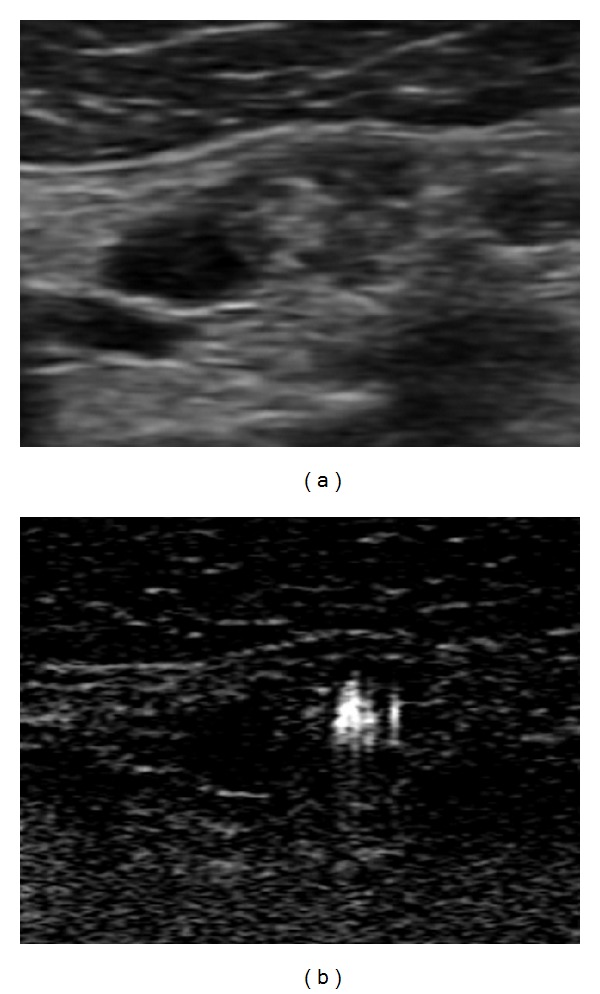
Metastatic lymph nodes at B-mode and BFI examination in patients with papillary thyroid cancer. The lymph node presents microcalcifications and multiple BFI-TS in the same place.

**Figure 3:**
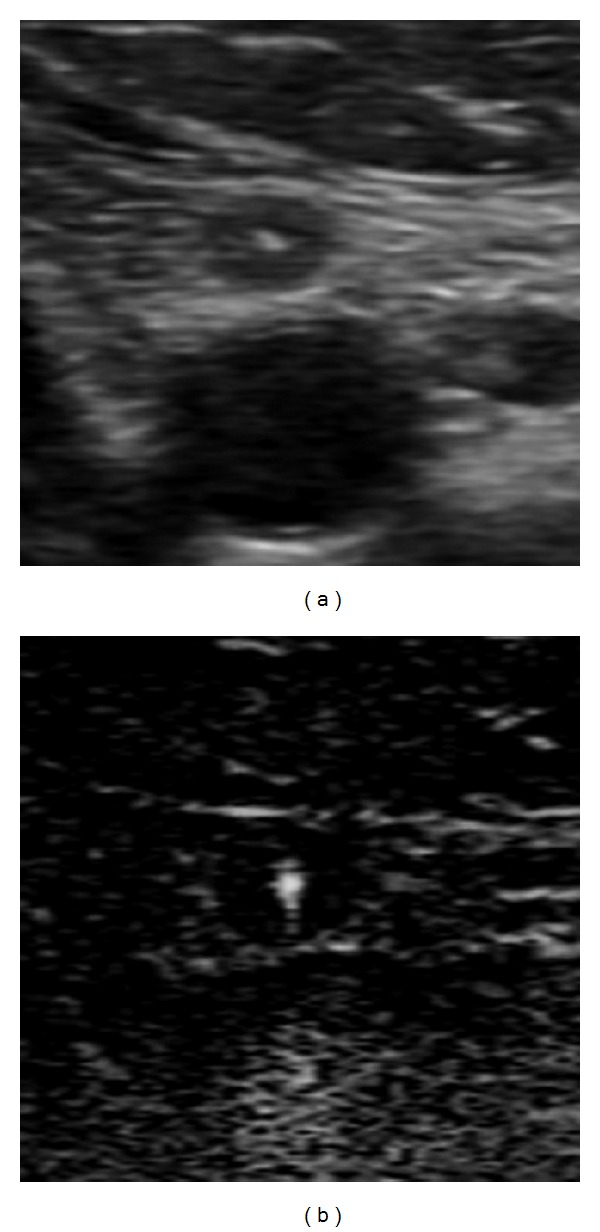
Small metastatic lymph node at B-mode and BFI examination in patients with papillary thyroid cancer. The lymph node presents BFI-TS without any suspect US features.

**Figure 4:**
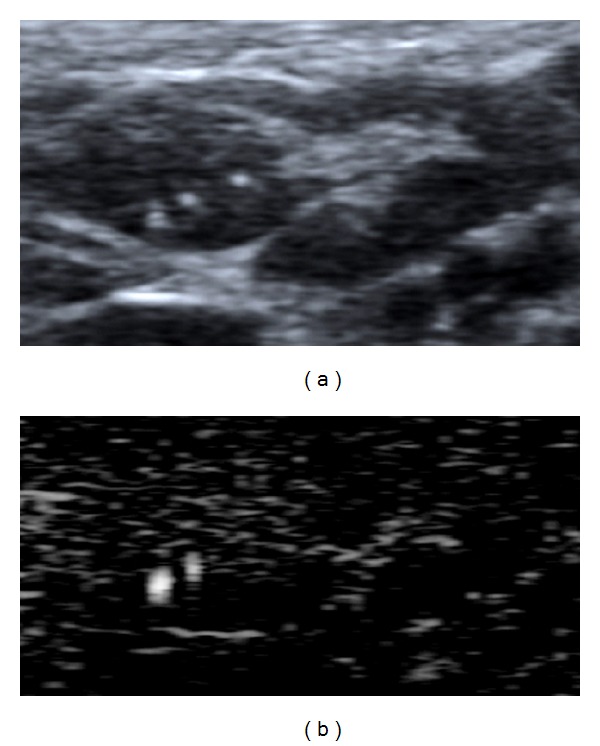
Focal metastasis in upper pole of lymph node at B-mode and BFI examination in patients with papillary thyroid cancer. The lymph node presents BFI-TS in the metastatic pole.

*The BFI-TS* had a higher specificity and sensitivity (resp., 99.7% and 80.9%) than conventional US features (Figure 2). The BFI-TS was positive in all LNs with calcification on US (93 LNs) and in 148 LNs (all metastatic) in which calcifications were not identified on US. The BFI-TS identified significantly more microcalcifications than B-mode US, and it also identified highly reflective and noncalcified structures such as colloidal crystals. This is confirmed by the histological findings of microcalcifications and colloidal crystals in the sites of BFI-TS. We detected BFI-TS in 6 metastatic LNs that were negative to the other conventional US features (Figure 3). Given its high specificity (99.7%), BFI-TS identifies better suspicious LNs that should be re-evaluated by surgery or US-guided FNAC (Figure 4). Therefore, the presence of BFI in addition to conventional US increases the diagnostic specificity for suspicious LNs (Table 2). As an example, the association of BFI-TS and absence of the hilum shows the best value of specificity, PPV, and diagnostic accuracy.

The BFI is an ultrasound technique that integrates conventional ultrasound but it does not replace it. When an LN presents at the least suspect ultrasound signs, it has to be studied also with the BFI since the positivity to BFI-TS gives evidence of its metastatic involvement with high diagnostic accuracy.

The techniques have several limits; namely, they can be affected by the pulsatility of the main neck vessel and by the deep places of examined LNs. These limits could explain the missed detection of 57 LNs (19%) that were metastatic at histological examination. The other limit is the presence of nonmetastatic LN calcifications; in fact, the BFI-TS was false-positive only in one LN with calcifications deriving from tuberculosis [36].

Overall, our results indicate that this technique can be applied to studies of cervical nodes in patients with PTC and that its sensitivity and specificity is higher than those of traditional US diagnostic techniques.

## 5. Conclusions

BFI is a promising imaging technique that can help in the differentiation of benign and metastatic neck LNs in patients with PTC. Our findings suggest that BFI can be helpful in the selection of suspicious neck LNs that should be examined cytologically or with open biopsy for accurate preoperative staging and individual therapy selection. A dedicated cervical US that includes nodal levels II–VI should be performed to detect nonpalpable LN metastases in patients undergoing surgical evaluation. However, longitudinal studies on a large population are required to verify the efficacy of BFI in the diagnosis of metastatic LNs. 

## Figures and Tables

**Table 1:** The diagnostic performance of sonographic criteria for metastatic lymph nodes in 83 patients with papillary thyroid cancer.

US features	Total lymph nodes (604)	Metastatic lymph nodes (298)	Sensitivity (%)	Specificity (%)	*P*	PPV	NPV
Round shape Short to long axis (diameter ratio > 0.5)	183	155	52	90.8	*χ* ^2^ 131.3 *P* 0.0000	84.7	66

Abnormal echogenicity	290	244	81.9	85	*χ* ^2^ 270.3 *P* 0.0000	84.1	82.8

Absence of the hilum	400	274	91.9	58.8	*χ* ^2^ 174 *P* 0.0000	68.5	88.2

Calcification	94	93	31.2	99.7	*χ* ^2^ 109.5 *P* 0.0000	98.9	59.8

Cystic change	63	63	21.1	100	*χ* ^2^ 72.2 *P* 0.0000	100	56.6

Peripheral vascularity	238	142	47.6	68.6	*χ* ^2^ 16.7 *P* 0.0000	59.7	57.4

BFI-TS	242	241	80.9	99.7	*χ* ^2^ 407.9 *P* 0.0000	99.6	84.2

US: Ultrasound; LNs: lymph nodes; PPV: positive predictive value; NPV: negative predictive value; BFI-TS: B-flow imaging twinkling sign.

**Table 2:** The diagnostic performance of the combination of each conventional ultrasound signs with the BFI-TS.

Combined US features	Total lymph nodes (604)	Metastatic lymph nodes (298)	Sensitivity (%)	Specificity (%)	*P*	PPV	NPV
Round shape Short to long axis (diameter ratio > 0.5) + BFI-TS	126	125	41.9	99.7	*χ* ^2^ 158.4 *P* 0.0000	99.2	63.8

Abnormal echogenicity + BFI-TS	200	200	67.1	100	*χ* ^2^ 307 *P* 0.0000	100	75.7

Absence of the hilum + BFI-TS	221	221	74.2	100	*χ* ^2^ 357.9 *P* 0.0000	100	78.9

Calcification + BFI-TS	92	92	30.9	100	*χ* ^2^ 111.4 *P* 0.0000	100	59.8

Cystic change + BFI-TS	61	61	20.5	100	*χ* ^2^ 69.7 *P* 0.0000	100	56.4

Peripheral vascularity + BFI-TS	117	116	39.9	99.7	*χ* ^2^ 144 *P* 0.0000	99.1	62.6

US: ultrasound; LNs: lymph nodes; PPV: positive predictive value; NPV: negative predictive value; BFI-TS: B-flow imaging twinkling sign.
